# circMAP3K4 regulates insulin resistance in trophoblast cells during gestational diabetes mellitus by modulating the miR-6795-5p/PTPN1 axis

**DOI:** 10.1186/s12967-022-03386-8

**Published:** 2022-04-21

**Authors:** Runyu Du, Na Wu, Yu Bai, Lei Tang, Ling Li

**Affiliations:** Department of Endocrinology, Shengjing Hospital of China Medical University, Shenyang, China

**Keywords:** Circular RNA, circMAP3K4, Gestational diabetes mellitus, miR-6795-5p, Insulin resistance, Trophoblast cell, PTPN1

## Abstract

**Background:**

Insulin resistance (IR) during gestational diabetes mellitus (GDM) has been linked to dysregulated insulin-PI3K/Akt pathway. A defective insulin-PI3K/Akt pathway and dysregulated circular RNA (circRNA) levels have been observed in the placentas of patients with GDM; however, the mechanisms underlying this association remain unclear.

**Methods:**

circRNAs potentially associated with GDM were selected through bioinformatics analysis and initially identified by quantitative real-time PCR (qPCR) in 9 GDM patients and 9 healthy controls, of which circMAP3K4 was further validated in additional 84 samples by qPCR. circMAP3K4 identity and localization were verified. Pearson correlation analysis was applied to evaluate the correlation between circMAP3K4 expression in the placental tissues of GDM patients and IR-related indicators. An IR model of trophoblasts was constructed using glucosamine. Interactions between miR-6795-5p and circMAP3K4 or *PTPN1* were confirmed using a dual-luciferase reporter assay. The circMAP3K4/miR-6795-5p/PTPN1 axis and key markers in the insulin-PI3K/Akt pathway in placentas and trophoblasts were evaluated through qRT-PCR, immunofluorescence, and western blotting. The role of circMAP3K4 in glucose metabolism and cell growth in trophoblasts was determined using the glucose uptake and CCK8 assay, respectively.

**Results:**

circMAP3K4 was highly expressed in the placentas of patients with GDM and the IR trophoblast model; this was associated with a dysregulated insulin-PI3K/Akt pathway. circMAP3K4 in the placentas of GDM patients was positively correlated with weight gain during pregnancy and time-glucose area under the curve of OGTT. circMAP3K4 and *PTPN1* could both bind to miR-6795-5p. miR-6795-5p and PTPN1 were downregulated and upregulated, respectively, in the placentas of GDM patients and the IR trophoblast model. circMAP3K4 silencing or miR-6795-5p overexpression partially reversed the decrease in glucose uptake, inhibition in cell growth, and downregulated IRS1 and Akt phosphorylation in IR-trophoblasts; this restoration was reversed upon co-transfection with an miR-6795-5p inhibitor or *PTPN1*.

**Conclusion:**

circMAP3K4 could suppress the insulin-PI3K/Akt signaling pathway via miR-6795-5p/PTPN1 axis, probably contributing to GDM-related IR.

**Supplementary Information:**

The online version contains supplementary material available at 10.1186/s12967-022-03386-8.

## Background

Gestational diabetes mellitus (GDM) refers to the situation when glucose intolerance is first detected or develops during pregnancy, and accounts for 86.4% of hyperglycemia cases in pregnancy [1, 2]. Globally, approximately 14% of pregnant women are affected by GDM, which leads to short-term and long-term complications both in mothers and offspring [3–5]. Although the precise etiological mechanisms underlying GDM remain unclear, insulin resistance (IR) is considered the hallmark of GDM [6]. During normal pregnancy, decreased insulin sensitivity in target tissues (such as fat tissue and skeletal muscle), which limits maternal glucose utilization and promotes hepatic glucose output, is conducive for fetal nutrition supply [7–10]. When compensatory insulin secretion from pancreatic β-cells is insufficient to maintain circulating glucose concentrations in response to insulin resistance, GDM occurs in pregnant women [2]. Insulin sensitivity in individuals with GDM is approximately 40% lower than that in normal controls during the last trimester of pregnancy; this may be related to a dysregulated insulin signaling pathway [6]. The insulin-phosphatidylinositol-3-kinase (PI3K)/Akt pathway is vital for insulin signal transduction; this pathway is activated by insulin receptor substrate (IRS) phosphorylation, which in turn activates multiple downstream cascades, thereby promoting cellular glucose uptake and regulating cell growth [11]. Dysregulated insulin-PI3K/Akt pathway has been observed in the fat tissues, skeletal muscles as well as placentas of GDM patients and animals [6, 12]. As the main organ for nutrient transportation and oxygen exchange between the fetal and maternal systems, the placenta is regarded as a "metabolic mirror" that reflects both maternal and fetal metabolic status [6, 13, 14]. Insulin-PI3K/Akt pathway dysregulation in the placenta of patients with GDM is synchronized with that in other insulin-sensitive tissues, thereby mirroring the maternal insulin sensitivity. Therefore, clarifying the mechanisms of the insulin-PI3K/Akt pathway in the placentas of GDM patients would help improve the understanding of GDM-related IR.

As a noncoding RNA (ncRNA) subclass, circular RNAs (circRNAs) have a covalently closed loop structure with neither 5ʹ caps nor 3ʹ polyadenylated tails, and exhibit better stability than linear RNAs [15]. Numerous studies have demonstrated circRNA involvement in a myriad of cellular signaling pathways, including IR pathogenesis [16, 17]. With the recent increase in the application of high-throughput sequencing, dysregulated circRNAs have been discovered in large quantities in the placentas of GDM patients. For example, Wang et al. [18] detected 46 differentially expressed circRNAs in the placentas of GDM patients, while Yan et al. [19] identified 482 abnormally expressed circRNAs in the placental villi of GDM patients. This circRNA dysregulation in the placentas of GDM patients might be significantly involved in placental signaling pathways; however, few studies have focused on the detailed functional evaluation of single circRNAs. Recently, circ_0005243 was determined to be significantly downregulated in the placenta and peripheral blood of GDM patients [20]. Moreover, circ_0005243 knockdown induced an increase in the secretion of pro-inflammatory factors (tumor necrosis factor-α and interleukin 6) in trophoblast cells, reduced β-catenin expression and increased nuclear factor-kappaB (NF-κB) p65 nuclear translocation. Even so, the detailed regulatory mechanism of circ_0005243 in the NF-κB pathway has not been determined.

circRNAs function at multiple levels, including the indirect regulation of gene expression by acting as a competitive endogenous RNA (ceRNA) of corresponding microRNAs (miRNAs) [15, 21]. Several studies have shown that differentially expressed miRNAs in the placentas of GDM patients can participate in the regulation of placental signaling pathways and affect glucose uptake, biological behavior, and the cell cycle in trophoblast cells. For example, miR-140-3p was upregulated in the placentas of GDM patients, and was found to contribute to insulin signaling pathway impairment by directly targeting the insulin receptor and the insulin-like growth factor 1 receptor, inhibiting glucose uptake in HTR-8/SVneo and human umbilical vein endothelial cells [12]. At the same time, miR-9 and mir-22 expression levels were decreased in the placentas of GDM patients; miR-9 and mir-22 depletion promoted glucose uptake and lactate secretion in primary syncytiotrophoblast and HTR-8/SVneo cells by targeting glucose transporter 1 and hexokinase-2 [22]. Based on the ceRNA theory, circRNA may contribute to the regulation of placental signaling pathways through miRNA sponging and the subsequent reversal of its repressive effects on downstream genes.

In this study, we aim to identify the aberrant circRNAs in the placentas of GDM that are closely related to GDM-related IR and evaluate the effects of selected circRNA on GDM-related IR in trophoblast cells through competitive endogenous RNA mechanisms.

## Materials and methods

### Bioinformatics analysis

**Fig. 1:**
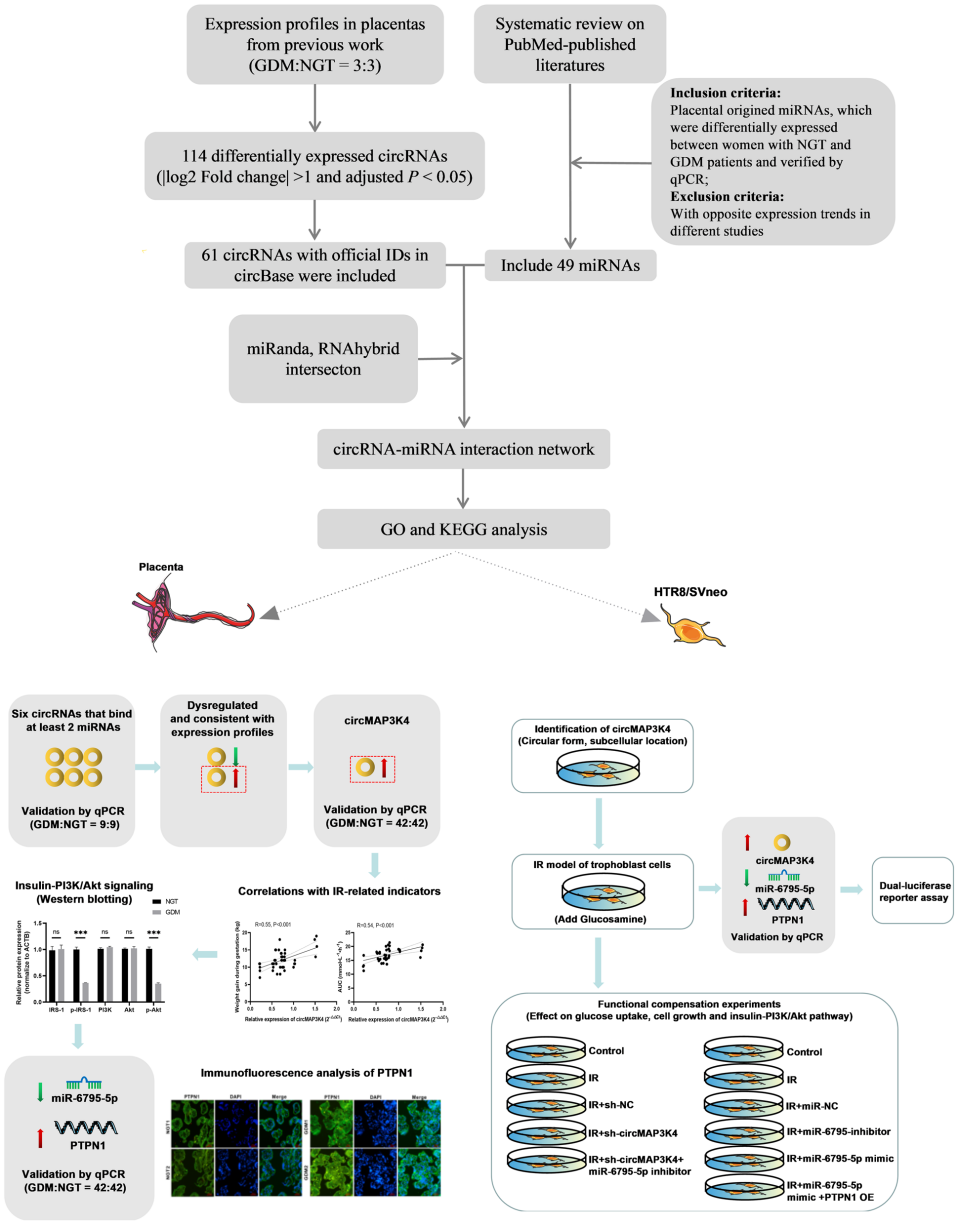
Schematic overview of the work flow

The work flow is shown in Fig. 1. Human circRNA microarray data were generated from one of our previous studies [23]. RNA was extracted from the placentas of three women with normal glucose tolerance (NGT) and three GDM patients. To identify differentially expressed circRNAs, we analyzed patient profiles using the “limma” function of the R package. |log2 Fold change|> 1 and adjusted *P*-value < 0.05 were retained as cut-off criteria. circRNAs with official IDs in the circBase database were retained for further analysis.

According to the ceRNA theory, circRNA can indirectly regulate target genes by sponging miRNA. We conducted a systematic review on PubMed-published data. The placental sourced miRNAs, which were differentially expressed between women with NGT and GDM patients and have been verified by quantitative real-time polymerase chain reaction (qPCR), were included for analysis. Moreover, those miRNAs that showed opposite expression patterns in different studies were excluded.

Interactions between the differentially expressed circRNAs and miRNAs were predicted using the miRanda (https://www.miranda.org/) and RNAhybrid (http://bibiserv.techfak.uni-bielefeld.de/rnahybrid/) tools. Results obtained using the miRanda and RNAhybrid tools were intersected. The Cytoscape 3.7.2 software (http://www.cytoscape.org/) was used to visualize the interactions.

Gene Ontology (GO) terms and pathways in the Kyoto Encyclopedia of Genes and Genomes (KEGG) were analyzed using the mirpath v.3 module of the DIANA tools software (http://diana.imis.athena-innovation.gr/DianaTools) to assess the underlying biological processes and pathways of circRNAs through their interactions with miRNAs.

### Participants and biopsy collection

All human experiments followed the Helsinki Guidelines and this study was approved by the Ethics Committee of Shengjing Hospital of China Medical University (2016PS360K). According to the Chinese Current Care Guidelines for GDM [24], GDM was diagnosed if one or more of the following criteria in a 75-g oral glucose tolerance test (OGTT) were presented at any time during pregnancy: 5.1 mmol/L ≤ fasting plasma glucose (FPG) < 7.0 mmol/L, 1-h plasma glucose (1hPG) ≥ 10.0 mmol/L, or 8.5 mmol/L ≤ 2-h plasma glucose (2hPG) < 11.1 mmol/L. The control group was matched to the GDM group through a 1:1 pattern according to age and pre-pregnancy body mass index (BMI). Women who met the criteria as follows were excluded: (1) younger than 18 years old, (2) history of diabetes, (2) infective or inflammatory diseases, (4) hypertension, (5) chronic diseases (thyroid dysfunction, cardio-cerebrovascular diseases, renal failure, etc.), (6) multiple pregnancies, or (7) used assistive reproductive technology. Finally, 51 patients with GDM and 51 pregnant women with NGT were recruited from Shengjing Hospital of China Medical University. After cesarean section, placental tissues were collected and stored at − 80 ℃. Nine pairs of samples were used for initial verification of the candidate circRNAs selected by bioinformatics, and the remaining 42 pairs were used for further validation of circMAP3K4. The maternal information and data on variable adverse neonatal outcomes were also collected.

### Total RNA and genomic DNA extraction

Extraction of cytoplasmic and nuclear RNA was performed using the PARIS™ system (Invitrogen, Carlsbad, CA, USA). TRIzol reagent (Invitrogen) was used to extract total RNA from placental tissues or cells following the manufacturer’s instructions. RNA purity and concentration were evaluated based on OD 260/280 readings performed using a NANO 2000 Spectrophotometer (Thermo Fisher Scientific, Waltham, MA, USA). Genomic DNA (gDNA) extraction was performed using the Genomic DNA Extraction Kit (Takara, Dalian, China).

### Quantitative real-time polymerase chain reaction

cDNA was generated from RNA using the BioTeke super RT kit (Bioteke, Beijing, China) according to the manufacturer’s protocol. qPCR was carried out using a SYBR GREEN mastermix (Solarbio, Beijing, China). PCR products were subjected to Sanger sequencing. Data were analyzed using the 2^−ΔΔCt^ method and normalized to the expression levels of beta-actin (*ACTB*) or U6 small nuclear RNA. All primers are described in Additional file 1: Table S1.

### Western blotting

Total protein was extracted from tissues and cells using the RIPA buffer (Shanghai, China), separated by SDS–PAGE, and then transferred to polyvinylidene difluoride membranes (PVDF, Millipore, Billerica, MA, USA). After the PVDF membranes were blocked with 5% skimmed milk, they were incubated overnight at 4 °C with primary antibodies anti-PTPN1 (tyrosine-protein phosphatase non-receptor type 1), anti-IRS1, anti-p-IRS1 (S307), anti-PI3K, anti-Akt, and anti-p-Akt (Ser473). Subsequently, the membranes were incubated with secondary antibodies for 2 h at room temperature. ACTB was used as the internal standard. Information about the antibodies is shown in Additional file 2: Table S2.

### Immunofluorescence analysis

Placental sections were incubated with primary anti-PTPN1 antibodies at 37 °C for 1 h and overnight at 4 °C. After incubation with fluoresce-labelled secondary antibodies and DAPI staining for nuclear counterstaining, images were taken using a microscope. Antibodies are listed in Additional file 2: Table S2.

### Cell culture and transfection

The human extravillous trophoblast cell line, HTR-8/SVneo, was purchased from the Chinese Academy of Sciences (Shanghai, China). Cells were cultured in RPMI-1640 (Gibco, Grand Island, NY, USA) supplemented with 10% fetal bovine serum (FBS, Hyclone, Logan, UT, USA) at 37 °C in 5% CO_2_. To induce insulin resistance, HTR-8/SVneo cells were incubated with 18 mM glucosamine for 18 h in serum-free medium [25, 26]. circMAP3K4 short hairpin RNA (shRNA) (sh-circMAP3K4) and shRNA negative control (sh-NC) were synthesized by Sangon company (Shanghai, China). The miR-6795-5p mimic, miR-6795-5p negative control (miR-NC), miR-6795-5p inhibitor, miR-6795-5p inhibitor negative control, and the overexpression plasmid (PTPN1 OE), were generated by GenePharma (Shanghai, China). HTR-8/SVneo cells were seeded in 6-well plates and cell transfections were performed using lipofectamine 3000 according to the manufacturer’s instructions.

### Ribonuclease R (RNase R) treatment

To identify the circularity, RNase R treatment was performed for 20 min at 37 °C using 3 U/mg RNase R (Lucigen, Middleton, WI, USA). Then, the resulting RNA was purified and analyzed by qPCR.

### Fluorescence in situ hybridization (FISH)

FISH was carried out using a fluorescent in situ hybridization kit (Promega, Beijing, China) to determine the subcellular localization of circMAP3K4 and miR-6795-5p. Specific probes for circMAP3K4 (cy3-labelled) and miR-6795-5p (FAM-labelled) (Servicebio, Wuhan, China) were used for in situ hybridization, and cell nuclei were simultaneously stained by DAPI. Images were taken using a Nikon Eclpse Ti-sr microscope (Nikon, Japan).

### Dual-luciferase reporter assay

The circMAP3K4 and *PTPN1* sequences containing the predicted potential miR-6795-5p wild-type (WT) binding sites or mutant (MUT) sites were subcloned into pmirGLO Dual-luciferase vectors (GenePharma). Then, HEK-293 cells were co-transfected with 500 ng of constructed dual-luciferase vectors and 30 nM miR-6795-5p mimic or miR-NC using Lipofectamine™ 2000 (Invitrogen). After 24 h of transfection, the cells were harvested and analysed according to the manufacturer’s instructions. The luciferase activities were normalised to the Renilla luciferase activity of the internal control using a dual luciferase assay kit (Promega).

### Glucose uptake assay

Glucose uptake in cells was evaluated using a 2-deoxyglucose (2-DG) Uptake Assay Kit (Biovision, Milpitas, CA, USA) following the manufacturer’s instructions. As with glucose, 2-DG can be uptaken by glucose transporters and metabolized to 2-DG-6-phosphate (2-DG6P), which cannot be further metabolized. The accumulated 2-DG6P, which is directly proportional to 2-DG uptake by cells, can be calculated to evaluate the glucose uptake ability. Briefly, 1 × 10^5^ HTR-8/Svneo cells were seeded in a 96-well plate and treated with desired method. Treated cells were starved for glucose by preincubating with 100 µL Krebs–Ringer-Phosphate-HEPES buffer containing 2% BSA for 40 min, followed by incubation with 10 µL of 10 mmol/L 2-DG. Glucose uptake were measured at OD412nm and calculated against a 2-DG6P standard curve during each experiment.

### Cell growth assay

Cell growth was determined by the cell counting kit-8 (CCK8) assay (Absin, Shanghai, China). According to the manufacturer's instructions, HTR-8/Svneo cells treated with desired method were seeded in 96-well culture plates overnight. At 24, 48 and 72 h, cells were interacted with 10 µL CCK8 solution for 1 h. The absorbance at a wavelength of 450 nm were then analyzed.

### Statistical analysis

Data were statistically analyzed using GraphPad Prism 9. Variables are presented as mean ± standard deviation or as number (percentage). The continuous variables were analyzed using Student’s t-test or One-way analysis of variance (ANOVA) followed by Bonferroni post-hoc analysis. The chi-square test was used to compare categorical variables. Spearman’s rho was used to determine the correlation between different variables. *P* < 0.05 was considered statistically significant. All reactions in vitro were performed in triplicate.

## Results

### Differentially expressed circRNA-miRNA interactions and enrichment analysis

**Fig. 2:**
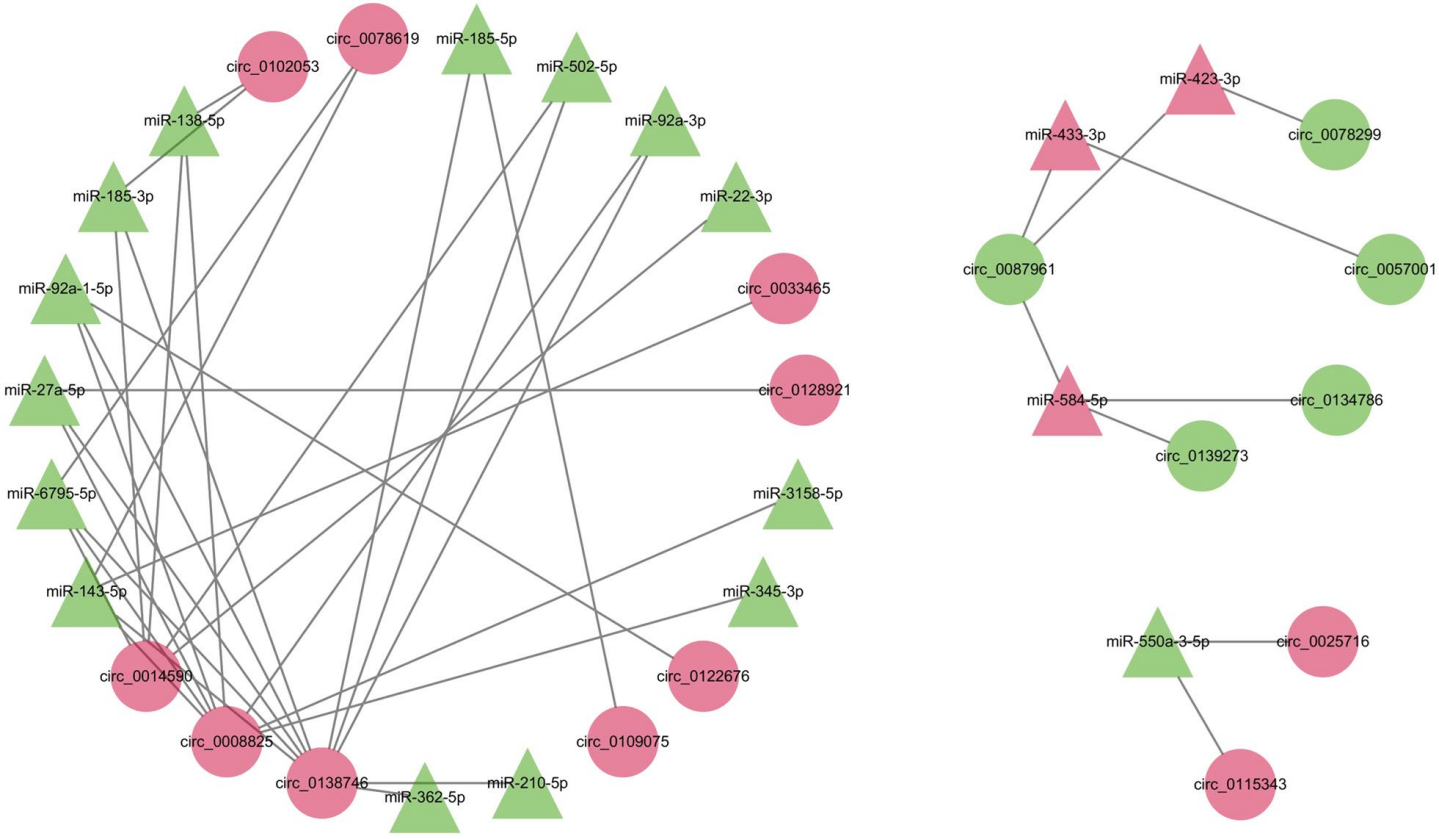
Interactions of the differentially expressed circRNA and miRNAs in the placentas of patients with gestational diabetes mellitus. circRNAs and miRNAs are represented by circles and triangles, respectively. The red and green colors represent up-regulation and down-regulation, respectively

As shown in Fig. 1, 114 circRNAs were differentially expressed in the placentas of GDM patients. Sixty-one circRNAs with corresponding circBase IDs, of which 28 were upregulated and 33 were downregulated, were retained for subsequent analyses. The presence of 49 miRNAs was pooled from the published data, of which 20 were upregulated and 29 were downregulated. The list of the included circRNAs and miRNA is shown in Additional file 3: Table S3. After prediction by miRanda and RNAhybrid, the differentially expressed circRNA-miRNA interaction network was constructed and visualized, and consisted of 16 circRNA nodes, 18 miRNA nodes, and 40 edges (Fig. 2).

Enrichment analysis was performed to determine the potential roles of circRNAs in the interaction network. The GO analysis involved 21 biological process (BP) terms, 11 cellular component (CC) terms, and 11 molecular function (MF) terms. The main enriched GO terms in each domain were “gene expression,” “cellular protein modification process,” and “biosynthesis process” in BP (Additional file 4: Fig. S1A); “microtubule tissue center part,” “lysosomal lumen,” and “nucleoplasm” in CC (Additional file 4: Fig. S1B); “transmembrane transporter activity,” “enzyme regulatory activity,” and “protein binding transcription factor activity” in MF (Additional file 4: Fig. S1C). A total of 70 KEGG pathways were enriched, and the altered circRNAs may also have affected several vital pathways involved in IR during pregnancy and GDM progression, including the insulin signaling pathway, the rapamycin pathway, and the adenosine monophosphate-activated protein kinase pathway (Additional file 4: Fig. S1D). A previous study of our team directly performed functional analysis for circRNAs [23]. Comparison was conducted according to whether circRNAs function in a miRNA-dependent manner or not, and 7 KEGG pathways were coincidenced (Additional file 5: Fig. S2). circRNAs may play more roles in the miRNA-dependent manners.

circRNAs that bind ≥ 2 miRNAs in their interaction networks (including circ_0138746, circ_0008825, circ_0014590, circ_0078619, circ_0102053, and circ_0087961) were initially validated in 18 placental tissues by qPCR. circ_0078619 and circ_0087961 were dysregulated in placentas of patients with GDM as compared to those of the controls (Additional file 6: Fig. S3), consistent with the sequencing results.

### circMAP3K4 was upregulated in GDM placentas and IR trophoblast model

**Fig. 3:**
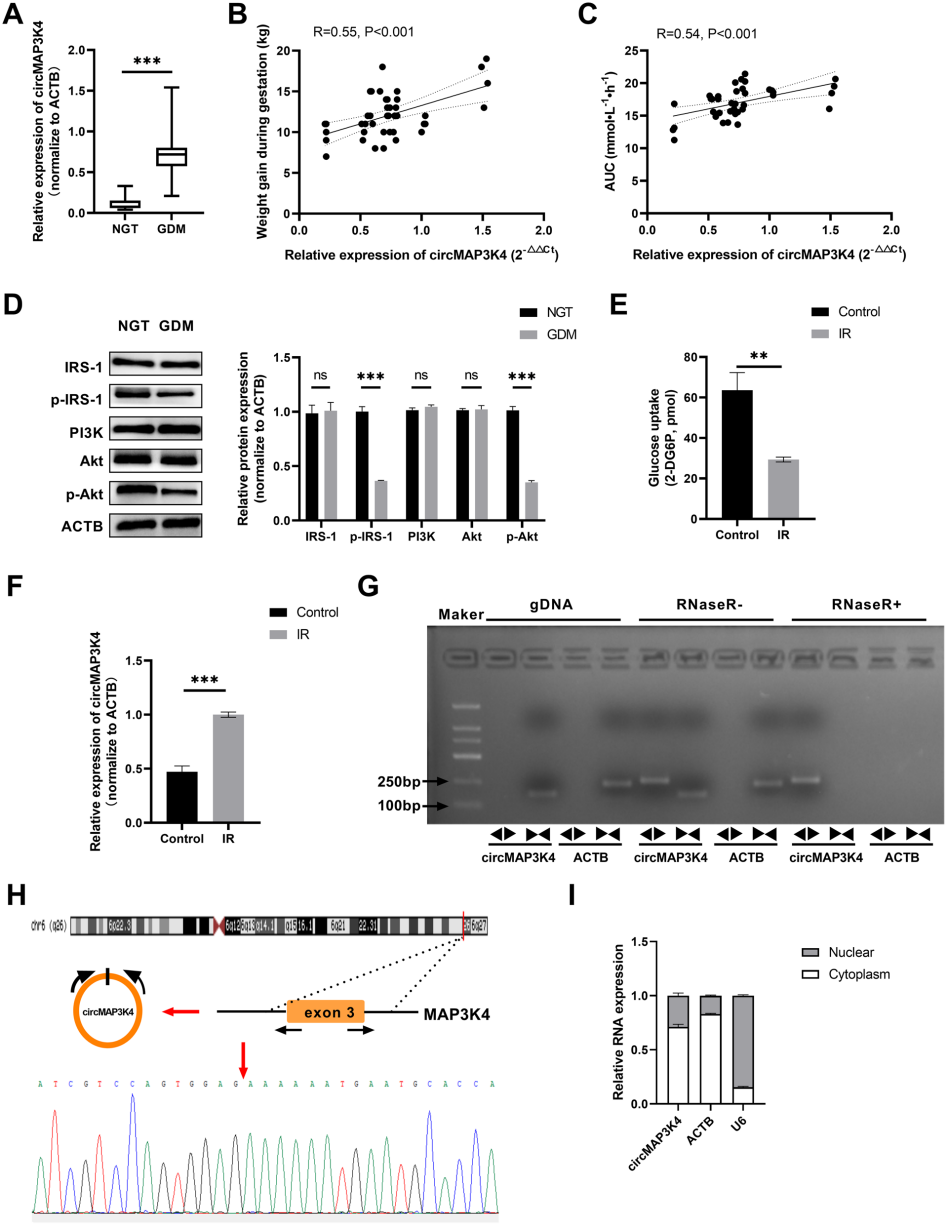
Identification of circMAP3K4 in GDM placentas and IR trophoblast model. **A** circMAP3K4 expression in the GDM (n = 42) and NGT (n = 42) groups as determined by qPCR. **B**, **C** circMAP3K4 expression in the placentas of GDM patients (n = 42) was positively correlated with weight gain during pregnancy and time-glucose AUC of the OGTT. **D** Insulin-PI3K/Akt signaling pathway markers were evaluated by western blotting in the GDM and NGT groups; the representative images of western blotting were displayed. **E** Construction of the IR model of HTR-8/SVneo cells. Glucose uptake ability was significantly decreased in IR group as compared to control cells. **F** circMAP3K4 expression in HTR-8/SVneo cells in the IR and control groups as determined by qPCR. **G** circMAP3K4 could resist digestion by RNase R, and circMAP3K4 was amplified to cDNA but not to gDNA using divergent primers. **H** Sanger sequence examined the circular splicing site of circ MAP3K4. **I** qRT-PCR analysis of circMAP3K4 in either the cytoplasm or the nucleus in HTR8/SVneo cells. The cell experiment was repeated three times. *P < 0.05, **P < 0.01, ***P < 0.001, ns: no significant difference

We focused our subsequent analyses on circ_0078619, which was upregulated. It is derived from exon 3 of mitogen activated protein kinase saucer 4 (MAP3K4) located on chromosome 6 (circbase: 161469647–161471011). Hereafter, we refer to it as circMAP3K4. We further determined circMAP3K4 expression in placental tissues in a larger sample size (tissues of 42 women with NGT and 42 GDM patients). As shown in Table 1, results of OGTT and HbA1c for the GDM group were significantly higher than those for the control group. There were no significant differences in maternal age, pre-pregnancy BMI, neonatal indexes, and placental weight between control and GDM pregnancies. Women with GDM were more likely to gain more weight during gestation and shorter gestational weeks compared with control subjects. circMAP3K4 levels were significantly higher in the GDM group than in the NGT group (Fig. 3A), and were positively correlated with weight gain during pregnancy (Fig. 3B) and time-glucose area under the curve (AUC) in the OGTT (Fig. 3C). Morover, p-IRS1 and p-Akt expression significantly decreased in GDM patients (Fig. 3D).

**Table 1:** Clinical characteristics of patients included in the study

Parameter	GDM (n = 42)	NGT (n = 42)	P value
Maternal age (years)	33.36 ± 4.85	31.48 ± 4.71	0.075
Pre-pregnancy BMI (kg/m^2^)	24.41 ± 4.77	25.98 ± 5.26	0.156
Weight gain during pregnancy (kg)	12.10 ± 2.72	9.52 ± 2.98	0.000***
Gravidity history	2.38 ± 1.27	1.95 ± 1.31	0.131
Parity history	0.40 ± 0.54	0.36 ± 0.48	0.673
Gestational age (days)	268.10 ± 10.19	272.74 ± 6.63	0.016*
OGTT at diagnosis of GDM or at 24 ~ 28 weeks of gestation			
0 min glucose (mmol/L)	5.28 ± 0.56	4.52 ± 0.22	0.000***
60 min glucose (mmol/L)	10.10 ± 1.79	7.15 ± 1.24	0.000***
120 min glucose (mmol/L)	8.68 ± 1.67	6.28 ± 0.93	0.000***
AUC (mmol·L^−1^·h^−1^)	12.56 ± 1.53	17.02 ± 2.37	0.000***
FPG at delivery (mmol/L)	4.79 ± 0.68	4.24 ± 0.49	0.000***
HbA1c at delivery (%)	5.61 ± 0.56	5.32 ± 0.31	0.006**
Baby's sex (female) (n[n%])	22 (52.38)	20 (47.62)	0.663
Birth weight (g)	3445.48 ± 483.68	3374.40 ± 486.82	0.504
Placental weight (g)	628.36 ± 103.55	613.95 ± 123.42	0.023
Apgar score (1 min)	9.88 ± 0.55	9.90 ± 0.37	0.817

GDM: gestational diabetes mellitus; BMI: body mass index; OGTT: oral glucose tolerance test; AUC: area under curve; FPG: fasting blood glucose; HbA1c: glycosylated hemoglobin

Data are expressed as mean ± standard deviation or n (n%), *P < 0.05, **P < 0.01, ***P < 0.001

To determine the detailed function of circMAP3K4 in GDM-related IR at cellular level, an HTR-8/SVneo cell IR model was constructed as described above. As IR signaling regulates glucose uptake in cells, glucose uptake was assessed in the IR model to confirm the occurrence of insulin resistance in the cells. As shown in Fig. 3E, glucose uptake was significantly repressed in HTR 8/SVneo cells in the IR group as compared to those in the control group. Moreover, elevated circMAP3K4 levels were observed in HTR 8/SVneo cells in the IR group (Fig. 3F). Subsequently, circMAP3K4 could resist digestion by RNase R, and was only amplified to cDNA, not gDNA, by divergent primers (Fig. 3G). PCR products were confirmed by Sanger sequencing, and the sequence result could completely match with the back-spliced region of circMAP3K4 (Fig. 3H). Additionally, qRT-PCR analysis of nuclear and cytoplasmic RNAs indicated that circMAP3K4 was preferentially localized in the cytoplasm in HTR8/SVneo cells (Fig. 3I). Collectively, our data indicated that aberrant circMAP3K4 expression is correlated with GDM-related IR and a dysregulated insulin-PI3K/Akt signaling pathway.

### circMAP3K4 is a spong of miR-6795-5p

**Fig. 4:**
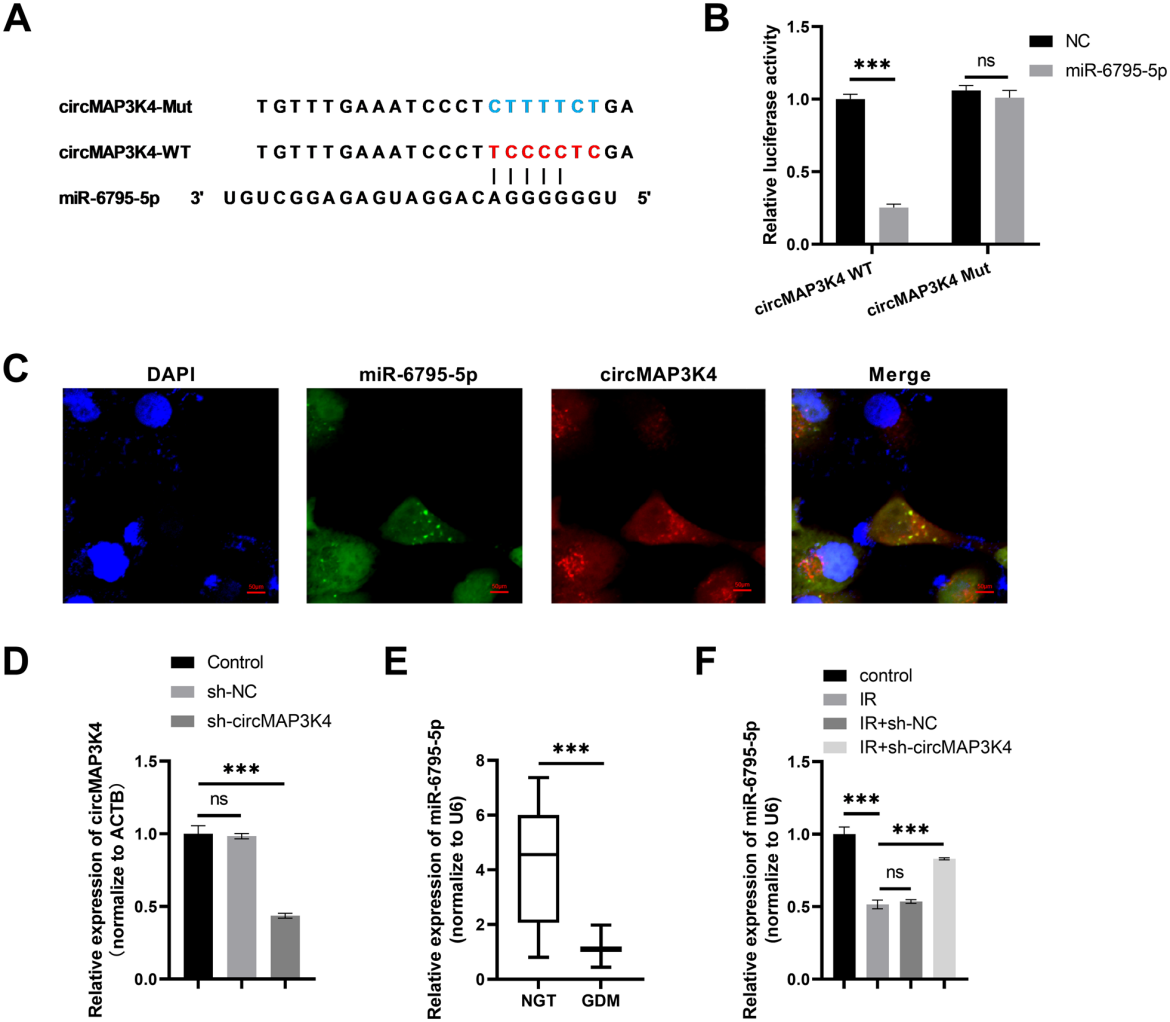
circMAP3K4 is a sponge of miR-6795-5p. **A** The predicted binding sites of circMAP3K4 on miR-6795-5p and mutant binding sites. **B** The dual-luciferase reporter assay was used to confirm the interaction between circMAP3K4 and miR-6795-5p. **C** Fluorescence in situ hybridization showed that circMAP3K4 and miR-6795-5p were co-located in the cytoplasm of HTR-8/SVneo cells; the red color represents circMAP3K4, while the green color represents miR-6795-5p. **D** circMAP3K4 expression was measured by qPCR to assess the transfection efficiency of sh-circMAP3K4. **E** miR-6795-5p expression in the GDM (n = 42) and NGT (n = 42) groups as determined by qPCR. **F** miR-6795-5p expression in HTR-8/SVneo cells in the different groups as determined by qPCR. The cell experiment was repeated three times. *P < 0.05, **P < 0.01, ***P < 0.001, ns: no significant difference

To explore the mechanism underlying circMAP3K4 activity in trophoblast cells, a search for the miRNA targets of circMAP3K4 was carried out using miRanda and RNAhybrid, and miR-6795-5p was found to carry circMAP3K4 binding sites (Fig. 4A). The dual luciferase reporter assay showed that miR-6795-5p overexpression could significantly inhibit wild-type circMAP3K4 luciferase activity but not that of the mutant (Fig. 4B). In addition, FISH analysis showed that circMAP3K4 and miR-6795-5p were co-expressed in the cytoplasm of HTR-8/SVneo cells (Fig. 4C). By measuring circMAP3K4 expression, we confirmed that its expression was inhibited in the sh-circMAP3K4 group as compared to the sh-NC group (Fig. 4D). Furthermore, miR-6795-5p expression was downregulated in the placental tissues of GDM patients (Fig. 4E) and in HTR8/SVneo cells in the IR group (Fig. 4F); at the same time, silencing circMAP3K4 induced an increase in miR-6795-5p expression (Fig. 4F).

### circMAP3K4 regulates IR in trophoblast cells by sponging miR-6795-5p

**Fig. 5:**
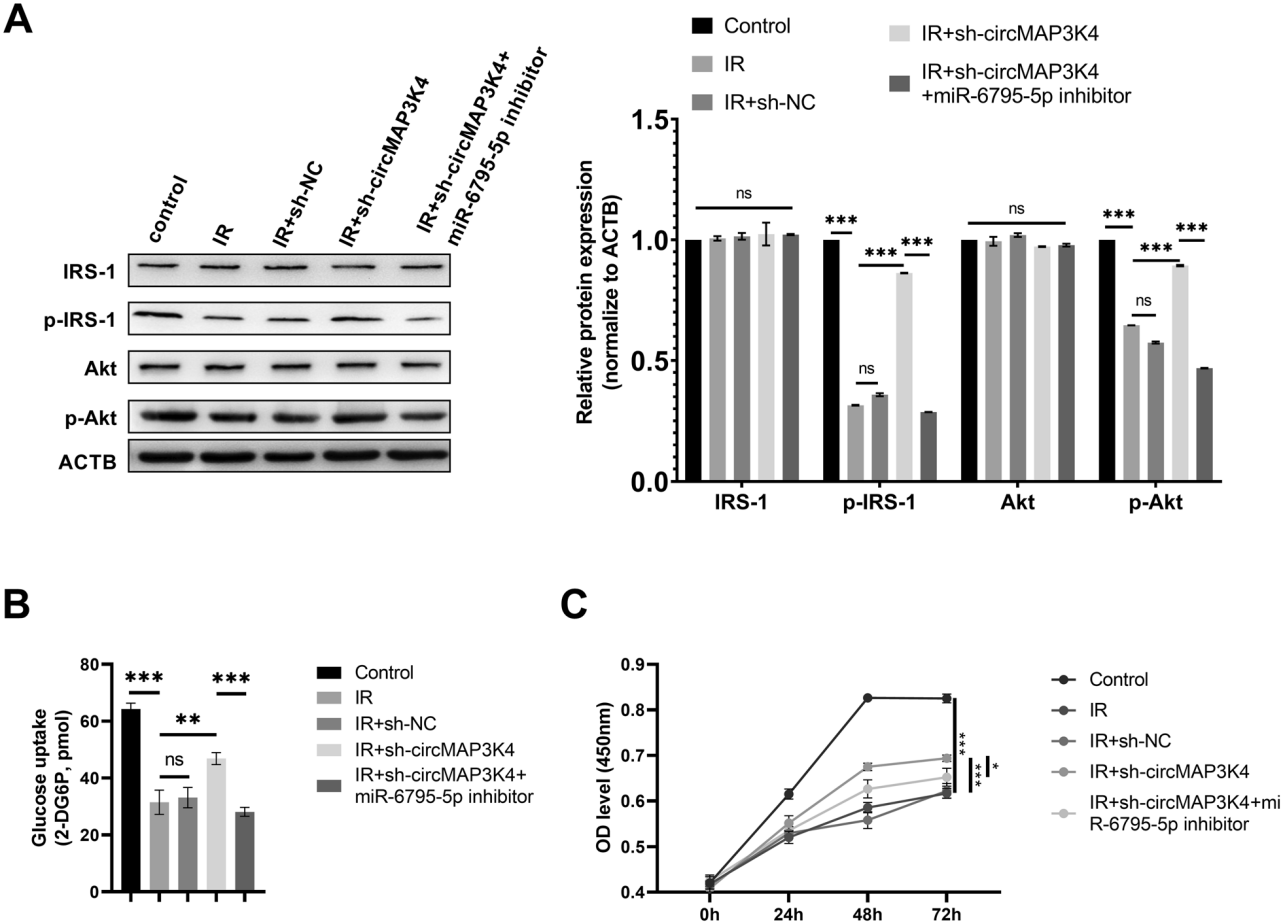
circMAP3K4 silencing partially restored insulin signaling in trophoblasts through miR-6795-5p. **A** The protein expression levels of IRS1, p-IRS1, Akt, and p-Akt were determined by western blotting, the representative images were displayed. **B** A glucose uptake assay was performed to determine the glucose uptake abilities of HTR-8/SVneo cells. **C** CCK8 assay was performed to determine the cell growth of HTR-8/SVneo cells. The cell experiment was repeated three times. *P < 0.05, **P < 0.01, ***P < 0.001, ns: no significant difference

To determine the role of circMAP3K4 in IR in trophoblast cells, we silenced its expression in IR model of HTR-8/SVneo cells. We observed IRS1 and Akt protein expression and phosphorylation in each group. p-IRS1 and p-Akt expression was decreased in the IR group, and circMAP3K4 silencing significantly promoted p-IRS1 and p-Akt expression; this effect could be nullified by co-transfection with the miR-6795-5p inhibitor (Fig. 5A). The decreased glucose uptake observed in the IR group was partially restored by circMAP3K4 knockdown, and co-transfection with sh-circMAP3K4 and a miR-6795-5p inhibitor induced a dysregulation in glucose uptake again (Fig. 5B). We further explored whether circMAP3K4 affects cell growth in trophoblast cells via miR-6795-5p targeting. The inhibited cell growth of trophoblasts induced by IR could be partially reversed by silencing circMAP3K4; this restoration was reversed upon co-transfection with an miR-6795-5p inhibitor ((Fig. 5C).

### miR-6795-5p binds to PTPN1

**Fig. 6:**
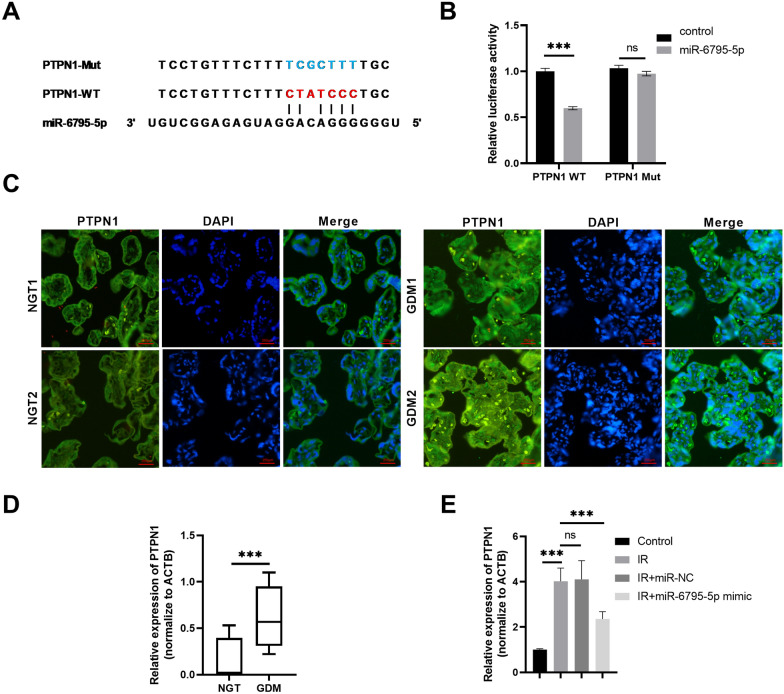
miR-6795-5p binds to *PTPN1*. **A** The predicted binding sites of *PTPN1* on miR-6795-5p and mutant binding sites. **B** The dual-luciferase reporter assay was used to confirm the interactions between *PTPN1* and miR-6795-5p. **C** Representative immunofluorescence of PTPN1 in the GDM and NGT groups. **D** PTPN1 mRNA expression in the GDM (n = 42) and NGT (n = 42) groups as determined by qPCR. **E** *PTPN1* expression in HTR-8/SVneo cells in the different groups was determined by qPCR. The cell experiment was repeated three times. *P < 0.05, **P < 0.01, ***P < 0.001, ns: no significant difference

As shown in Fig. 6A, based on the predictions of the DIANA-microT-CDS module in the DIANA tools, miR-6795-5p was found to carry binding sites for *PTPN1*. PTPN1 is a negative regulator of the insulin-PI3K/Akt signaling pathway [27, 28]. Subsequently, a decrease in luciferase activity in HTR-8/SVneo cells co-transfected with miR-6795-5p and PTPN1-WT confirmed the direct interaction between miR-6795-5p and *PTPN1* (Fig. 6B). PTPN1 mRNA and protein expression were upregulated in the placental tissues of GDM patients (Fig. 6C, D). Moreover, *PTPN1* was up-regulated in the HTR-8/SVneo cell IR model, and its expression significantly decreased following miR-6795-5p overexpression in IR-trophoblasts (Fig. 5E). These results indicated that miR-6795-5p could negatively regulate *PTPN1* mRNA expression.

### miR-6795-5p modulates IR in trophoblast cells through PTPN1

**Fig. 7:**
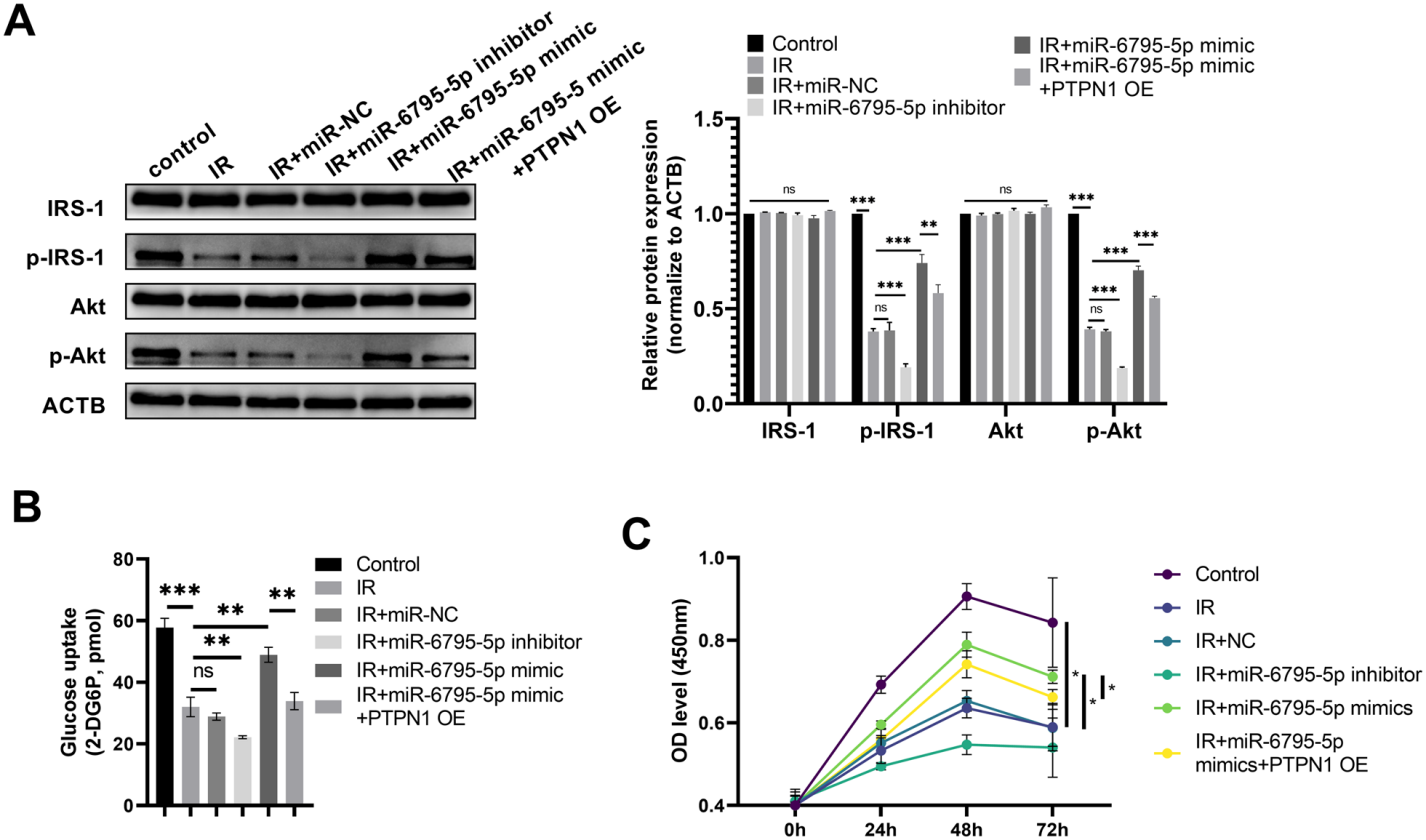
miR-6795-5p overexpression restored insulin resistance in trophoblasts by targeting *PTPN1*. **A** The protein expression levels of IRS1, p-IRS1, Akt, and p-Akt were determined by western blotting, the representative images were displayed. **B** A glucose uptake assay was performed to determine the glucose uptake abilities of HTR-8/SVneo cells. **C** CCK8 assay was performed to determine the cell growth of HTR-8/SVneo cells. The cell experiment was repeated three times. *P < 0.05, **P < 0.01, ***P < 0.001, ns: no significant difference

miR-6795-5p overexpression significantly rescued the decreased p-IRS1 and p-Akt expression induced by IR, and this was reversed by co-transfection with *PTPN1* and the miR-6795-5p mimic (Fig. 7A). Furthermore, a further decrease in p-IRS1 and p-Akt expression levels was observed following transfection with the miR-6795-5p inhibitor in the IR group (Fig. 7A). Glucose uptake ability was further reduced after transfection with the miR-6795-5p inhibitor in the IR group. The repression in glucose uptake was partially restored by miR-6795-5p overexpression; however, this effect could be reversed by co-transfection with *PTPN1* and the miR-6795-5p mimic (Fig. 7B). In addition, the inhibited cell growth of trophoblasts induced by IR could be partially reversed by miR-6795-5p overexpression; this restoration was reversed upon co-transfection with PTPN1 (Fig. 7C).

### circMAP3K4 regulates PTPN1 expression through miR-6795-5p

**Fig. 8:**
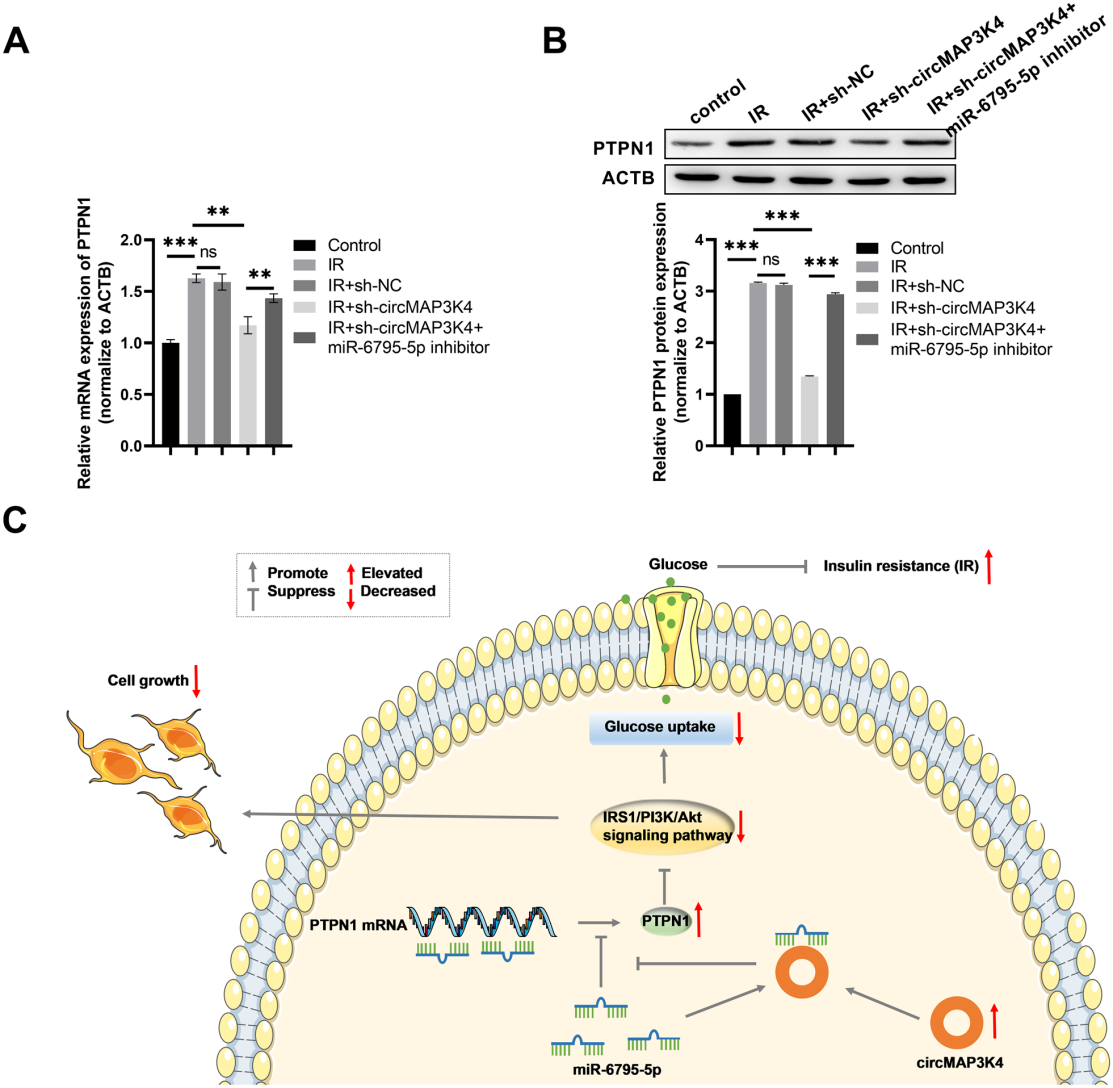
circMAP3K4 regulates PTPN1 expression through miR-6795-5p. **A** PTPN1 mRNA expression in HTR-8/SVneo cells in the different groups as determined by qPCR. **B** Western blot analysis of PTPN1 protein expression in different groups of HTR-8/SVneo cells, the representative images were displayed. **C** circMAP3K4 acts as a sponge of miR-6795-5p to modulate insulin resistance (IR) in trophoblast cells by regulating PTPN1 expression. The cell experiment was repeated three times. *P < 0.05, **P < 0.01, ***P < 0.001, ns: no significant difference

PTPN1 mRNA and protein expression levels in every HTR-8/SVneo cell group were measured by qPCR and western blotting. As shown in Fig. 8A, compared to cells in the IR group, relative *PTPN1* mRNA expression significantly decreased following circMAP3K4 silencing in HTR-8/SVneo cells in the IR-treated group, and this effect could be annulled by co-transfection with sh-circMAP3K4 and the miR-6795-5p inhibitor. Similar changes were observed at the protein level through western blotting (Fig. 8B). Therefore, we concluded that circMAP3K4 could regulate *PTPN1* expression by binding to miR-6795-5p, thereby regulating the insulin-PI3K/Akt signaling pathway and inhibiting glucose uptake in trophoblast cells, possibly contributing to GDM-related IR (Fig. 8C).

## Discussion

As the most common hyperglycemic manifestation during pregnancy [1, 2], GDM affects short-term and long-term health in mothers and offspring [3–5]; however, its pathological mechanism remains unclear. IR is recognized as a specific pathological feature of GDM [6], and could be partly due to insulin-PI3K/Akt signaling pathway impairment in insulin-sensitive tissues such as fat tissue and skeletal muscles [7–9]. The insulin receptor (INSR) is activated by insulin and the down-stream protein, IRS, is associated with tyrosine phosphorylation (p-IRS); phosphorylated tyrosine activates PI3K by binding to its SH2 domain, and activated PI3K in-turn stimulates Akt. Activated Akt (p-Akt) promotes cellular glucose uptake by regulating glucose transporter translocation to the cell membrane [11, 29]. The dysregulation of any of these processes results in IR.

Due to the "high efficiency" of INSR, only 10–20% of receptors can exert the maximum insulin binding effect. Thus, insulin signal transduction inhibition following receptor activation has attracted more attention [30]. IRS-1 and IRS-2 are the most studied substructures of IRS; IRS1 mainly promotes glucose uptake and utilization in peripheral tissues as it has similar effects to insulin, while IRS-2 is mainly involved in hepatic glycogen synthesis and gluconeogenesis [31].

Few studies have focused on evaluating defective insulin-PI3K/Akt signaling in the placentas of GDM patients [32, 33]. In this study, phosphorylation levels of IRS1 and Akt were decreased in the placentas of GDM patients, and this was consistent with the findings of a previous study [34]. No significant changes in PI3K protein expression were observed, possibly due to the fact that the action of PI3K is mainly related to conformational changes rather than to absolute protein expression levels. Moreover, the biological effect of PI3K occurs mainly through the conversion of Akt to p-Akt. We observed a decrease in IRS1 and Akt phosphorylation levels and reduced glucose uptake in the IR trophoblast cell model. Concurrently, the insulin-PI3K/Akt signaling pathway was dysregulated in the placentas of GDM patients and glucosamine-treated trophoblast cells, indicating an association between insulin-PI3K/Akt signaling pathway dysregulation in placental cells and maternal IR.

Increasing evidence has shown that circRNAs participate in the regulation of signaling pathways [15, 21, 35, 36], and are closely related to IR development [16, 17]. Numerous circRNAs have been found to be differentially expressed in the placentas of GDM patients [18, 19], indicating that aberrant circRNA expression may be involved in the dysregulation of signaling pathways in the placentas of GDM patients. However, few studies have focused on evaluating the effects of single circRNAs on defective signaling pathways in the placentas of GDM patients. Based on the results of our previous high-throughput sequencing [23] and bioinformatics analyses, we speculated that circMAP3K4 up-regulation might be associated with GDM pathogenesis. Through qPCR, we confirmed that circMAP3K4 was highly expressed in the placentas of GDM patients, and its expression was positively correlated with maternal weight gain during pregnancy and OGTT time blood glucose AUC. Maternal obesity and excessive weight gain can further aggravate insulin resistance during pregnancy, and are independent risk factors for GDM [37]. Time-glucose AUC in the OGTT reflects IR levels in GDM patients, and is associated with adverse perinatal outcomes in GDM patients and their offspring [38, 39]. Therefore, circMAP3K4 may be significantly associated with GDM-related IR, as well as insulin-PI3K/Akt signaling pathway dysregulation in placental tissues.

circRNAs can serve as upstream target mRNA regulators by competitively binding to miRNAs [15]. As mentioned, insulin-PI3K/Akt signal pathway dysregulation was observed in the placentas of GDM patients, and this reflects maternal IR levels. We speculated that circMAP3K4 targets the negative regulator of the insulin PI3K/Akt signaling pathway through miRNAs. PTPN1, also known as protein tyrosine phosphatase 1B, can dephosphorylate IRS-1, thereby affecting downstream cascade reactions and inhibiting signal transduction in the insulin-PI3K/Akt signaling pathway [27, 28]. Based on the results of our bioinformatics analyses, circMAP3K4 could regulate the insulin-PI3K/Akt pathway through the miR-6795-5p/PTPN1 axis. The function of circMAP3K4 has not yet been investigated. It was reported that miR-6795-5p was significantly downregulated in exosomes derived from the chorionic villi of GDM patients as compared to those from women with normal pregnancies; however, the specific functional role of miR-6795-5p in GDM was not clarified [40]. Several studies identified PTPN1, a major negative regulator for insulin receptor signaling, as an ideal therapeutic target for intervention in type 2 diabetes and obesity [27, 41, 42]. Genetic deletion or pharmacological inhibition of PTPN1 are able to maintain glucose homeostasis with about half the level of circulating insulin and relieved the insulin receptor phosphorylation in liver and muscle [43, 44]. Thus, the function of the circMAP3K4/miR-6795-5p/PTPN1 axis in the regulation of the insulin-PI3K/Akt signaling pathway in GDM patient placentas was investigated in this study. The dual-luciferase reporter assay confirmed that circMAP3K4 and *PTPN1* could both bind to miR-6795-5p. circMAP3K4 and miR-6795-5p were co-located in the cytoplasm of HTR-8/SVneo cells; thus, we speculated that circMAP3K4 might alter the biological functions of trophoblast cells by acting as an miR-6795-5p sponge. In addition, miR-6795-5p was down-regulated in GDM patient placental tissues and in the HTR-8/SVneo cell IR model, while *PTPN1* was up-regulated. Of note, our rescue experiments revealed that circMAP3K4 down-regulated insulin-PI3K/Akt signaling pathway in trophoblast cells through the miR-6795-5p/PTPN1 axis, thus inhibiting glucose uptake ability and cell growth of trophoblasts. Placenta might not be a main organ in regulating glucose metabolism in whole body during the pregnancy. However, placenta or trophoblast could partially influence glucose metabolism; trophoblast cell growth potency could embody the influence of the IR in placenta and explain the more frequent pregnancy failure and decreased placental efficiency (fetal weight divided by placental weight) in patients with GDM compared with normal controls.

Limitations were present in this study. In addition to placenta, circMAP3K4 is also abundant in several insulin-targeted tissues, such as skeletal muscle, liver and heart [45, 46]. Whether circMAP3K4/miR-6795-5p/PTPN1 axis plays the same role in major maternal insulin-sensitive tissues remains to be further verified. According to the prediction by circAtlas [47], we found that circMAP3K4 may bound to several N6-methyladenine (M6A) regulators, such as YTH domain-containing family protein 1–2 and insulin-like growth factor 2 mRNA-binding protein 1–3. Thus, the m6A modification occurring in circMAP3K4 can modify their secondary structure, splicing, degradation, or molecular stability, which can influence the expression of the circRNAs. Since microRNAs co-regulate molecules in a network. A previous study found that LINC01013 directly bound to miR-6795-5p and subsequently relieved FMNL3 in hepatocellular carcinoma cells [48]. As shown in Fig. 2, miR-6795-5p interacted with other 3 circRNAs in our miRNA-circRNA network. As for *PTPN1*, 68 experimentally validated miRNA-target interaction could be obtained based on TarBase [49]. Therefore, circMAP3K4/miR-6795-5p axis may not necessarily affect *PTPN1* as a whole. Thus, there may be a more complicated network regulation. Moreover, circRNAs function in different patterns, which are not mutually exclusive. According to the prediction by circAtlas [47] and circRNADb [50], circMAP3K4 may also function by interacting RNA binding proteins or encoding peptide. It is likely that there are additional mechanisms at play. Additionally, insulin treatment during GDM would certainly lead to more complexities. Insulin-like growth family and their receptors serve as vital molecules in mediating the key metabolic actions of insulin. The effect of circMAP3K4/miR-6795-5p/PTPN1 axis on trophoblasts after adding insulin-like growth factors or insulin remain to be assessed in the future.

## Conclusion

In conclusion, our study proposed that circMAP3K4 suppressed glucose uptake and cell growth of trophoblast cells during GDM via regulating the activity of insulin-PI3K/Akt pathway through the miR-6795-5p/PTPN1 axis, probably contributing to the development of GDM. These findings indicate a new insight into uncovering the pathogenesis of GDM-related IR, as well as a novel potential therapeutic target.

## Supplementary Information

**Additional file 1: Table S1.** Primer sequences.

**Additional file 2: Table S2.** Antibodies used in the study.

**Additional file 3: Table S3.** List of circRNAs and miRNAs included in the study.

**Additional file 4: Figure S1.** Enrichment analysis. Cluster diagrams of enriched biological processes (A), cellular components (B), and molecular functions (C) of the GO terms and KEGG pathways (D). The horizontal axis represents the pathway name, while the vertical axis represents the enriched miRNAs. The gradient color represents the log (*P*-value).

**Additional file 5: Figure S2.** The intersection of of KEGG pathways according to circRNAs function in a miRNA-dependent manner and in a miRNA-independent manner.

**Additional file 6: Figure S3.** circ_0078619 and circ_0087961 expression in the gestational diabetes mellitus (GDM, n = 9) and normal glucose tolerance (NGT, n = 9) groups as determined by qPCR. The cell experiment was repeated three times. *** P < 0.001.

## Data Availability

The processed data that support the findings of this study are available from the corresponding author upon reasonable request.

## Abbreviations

ACTB: Beta-actin
AUC: Area under curve
BMI: Body mass index
BP: Biological process
CC: Cellular component
ceRNA: Competitive endogenous RNA
circRNA: Circular RNA
CCK8: Cell counting kit-8
FISH: Fluorescence in situ hybridization
GDM: Gestational diabetes mellitus
GO: Gene Ontology
IR: Insulin resistance
IRS: Insulin receptor substrate
KEGG: Kyoto Encyclopedia of Genes and Genomes
MAP3K4: Mitogen-activated protein kinase 4
MF: Molecular function
miRNA: MicroRNA
ncRNA: Non-coding RNA
NF-κB: Nuclear factor-kappaB
NGT: Normal glucose tolerance
OGTT: Oral glucose tolerance test
PI3K: Phosphatidylinositol-3-kinase
PTPN1: Tyrosine-protein phosphatase non-receptor type 1
qPCR: Quantitative real-time polymerase chain reaction
shRNA: Short hairpin RNA
