# Supplementary material for: circMAP3K4 regulates insulin resistance in trophoblast cells during gestational diabetes mellitus by modulating the miR-6795-5p/PTPN1 axis
Source: J Transl Med. 2022 Apr 21;20:180. doi: 10.1186/s12967-022-03386-8 (PMC9022258; doi:10.1186/s12967-022-03386-8)
Supplement: Supplementary file 1 — Additional file 1: Table S1. Primer sequences. [file 12967_2022_3386_MOESM1_ESM.doc]

**Table S1. Primer sequences**

| **Gene** | **Forward** | **Reverse** |
| --- | --- | --- |
| **has_circ_0078619**  **(convergent)** | CAGGCACTCTGTTTGT | TGTCATCACCCTCATAC |
| **has_circ_0078619**  **(divergent)** | TGATTCTCTTGGCTGGGGAG | CCAGTGTCTTTATGTGGAGGC |
| **has_circ_0008825** | AGTGCCAGATTAGAGC | TCCTTCAGTCCACCTA |
| **has_circ_0138746** | GCCAACTGAGAACAAT | ATAGCCACCAAACATC |
| **has_circ_0014590** | TCACTGCCTTCTAACG | TGAATGAAGACGGATG |
| **has_circ_0102053** | ACTGGCTTCAGGTTTA | GGTATGTGACAGGAGGT |
| **hsa_circ_0087961** | TCAGCAAACAAGAAAA | ATTACAGGGATGGAAG |
| **miR-6795-5p** | TGGGGGGACAGGATGAGAG | GTGCAGGGTCCGAGGTATTC |
| **PTPN1** | TCCCTTTGACCATAGTCGGAT | GTGACCGCATGTGTTAGGCA |
| **β-actin** | CATGTACGTTGCTATCCAGGC | CTCCTTAATGTCACGCACGAT |
| **U6** | CTCGCTTCGGCAGCACA | AACGCTTCACGAATTTGCGT |
