# Supplementary material for: circMAP3K4 regulates insulin resistance in trophoblast cells during gestational diabetes mellitus by modulating the miR-6795-5p/PTPN1 axis
Source: J Transl Med. 2022 Apr 21;20:180. doi: 10.1186/s12967-022-03386-8 (PMC9022258; doi:10.1186/s12967-022-03386-8)
Supplement: Supplementary file 2 — Additional file 2: Table S2. Antibodies used in the study. [file 12967_2022_3386_MOESM2_ESM.doc]

**Table S2. Antibodies used in the study**

| **Name** | **Catalog number** | **Source** | **Dilution ratio** |
| --- | --- | --- | --- |
| **IRS1** | A0245 | Abclonal, Wuhan, China | (WB)1：1000 |
| **p-IRS1 (S307)** | AP0552 | Abclonal, Wuhan, China | (WB)1：1000 |
| **Akt (pan)** | 4691S | CST, USA | (WB)1：1000 |
| **p-Akt (Ser473)** | 4060S | CST, USA | (WB)1：1000 |
| **PTPN1** | 11334-1-AP | ProteinTech, Wuhan, China | (WB)1：2000  (IF)1：200 |
| **Goat Anti-Rabbit IgG** | 31460 | Thermo, USA | (WB)1：12000 |
| **FITC goat anti-rabbit IgG** | A0562 | Biyuntian, Shanghai, China | (IF)1：200 |
| **β-actin** | GB12001 | Servicebio, Wuhan, China | (WB)1：10000 |

WB: Western Blot, IF: Immunofluorescence
