# Supplementary material for: circMAP3K4 regulates insulin resistance in trophoblast cells during gestational diabetes mellitus by modulating the miR-6795-5p/PTPN1 axis
Source: J Transl Med. 2022 Apr 21;20:180. doi: 10.1186/s12967-022-03386-8 (PMC9022258; doi:10.1186/s12967-022-03386-8)
Supplement: Supplementary file 3 — Additional file 3: Table S3. List of circRNAs and miRNAs included in the study. [file 12967_2022_3386_MOESM3_ESM.doc]

**Table S3. List of circRNAs and miRNAs included in the study**

| **circRNA** | | **miRNA** | |
| --- | --- | --- | --- |
| **Up-regulated** | **Down-regulated** | **Up-regulated** | **Down-regulated** |
| hsa_circ_0010243 | hsa_circ_0010073 | hsa-miR-122-5p | hsa-miR-125b |
| hsa_circ_0011944 | hsa_circ_0011868 | hsa-miR-1323 | hsa-miR-138-5p |
| hsa_circ_0113603 | hsa_circ_0011995 | hsa-miR-136-5p | hsa-miR-143 |
| hsa_circ_0014590 | hsa_circ_0000174 | hsa-miR-137 | hsa-miR-145-3p |
| hsa_circ_0111367 | hsa_circ_0023692 | hsa-miR-182-3p | hsa-miR-185 |
| hsa_circ_0000261 | hsa_circ_0025487 | hsa-miR-186-5p | hsa-miR-203a-3b |
| hsa_circ_0023944 | hsa_circ_0029617 | hsa-miR-202-5p | hsa-miR-208a-3p |
| hsa_circ_0025716 | hsa_circ_0006327 | hsa-miR-210-3p | hsa-miR-21 |
| hsa_circ_0101676 | hsa_circ_0036413 | hsa-miR-222 | hsa-miR-210-5p |
| hsa_circ_0102053 | hsa_circ_0041258 | hsa-miR-29a-3p | hsa-miR-22 |
| hsa_circ_0033465 | hsa_circ_0050118 | hsa-miR-342-3p | hsa-miR-27a |
| hsa_circ_0103786 | hsa_circ_0056741 | hsa-miR-423-3p | hsa-miR-30d-5p |
| hsa_circ_0001982 | hsa_circ_0057001 | hsa-miR-433-3p | hsa-miR-3158-5p |
| hsa_circ_0005600 | hsa_circ_0061736 | hsa-miR-503 | hsa-miR-335-5p |
| hsa_circ_0109075 | hsa_circ_0066890 | hsa-miR-508-3p | hsa-miR-33a |
| hsa_circ_0109512 | hsa_circ_0067771 | hsa-miR-518d | hsa-miR-345-3p |
| hsa_circ_0120756 | hsa_circ_0068000 | hsa-miR-520h | hsa-miR-362 -5p |
| hsa_circ_0115343 | hsa_circ_0069341 | hsa-miR-584-5p | hsa-miR-369-3p |
| hsa_circ_0122676 | hsa_circ_0126072 | hsa-miR-657 | hsa-miR-411-5p |
| hsa_circ_0067908 | hsa_circ_0001459 | hsa-miR-98 | hsa-miR-451a |
| hsa_circ_0070571 | hsa_circ_0077706 |  | hsa-miR-4732-3p |
| hsa_circ_0008825 | hsa_circ_0078299 |  | hsa-miR-483-3p |
| hsa_circ_0128921 | hsa_circ_0079043 |  | hsa-miR-502-5p |
| hsa_circ_0132779 | hsa_circ_0134786 |  | hsa-miR-550a-3-5p |
| hsa_circ_0078619 | hsa_circ_0079632 |  | hsa-miR-574-3p |
| hsa_circ_0138746 | hsa_circ_0083425 |  | hsa-miR-6795-5p |
| hsa_circ_0139134 | hsa_circ_0087263 |  | hsa-miR-9 |
| hsa_circ_0089485 | hsa_circ_0008782 |  | hsa-miR-92a |
|  | hsa_circ_0139273 |  | hsa-miR-96-5p |
|  | hsa_circ_0087848 |  |  |
|  | hsa_circ_0087959 |  |  |
|  | hsa_circ_0087961 |  |  |
|  | hsa_circ_0089485 |  |  |
