# Supplementary figures and images for: circMAP3K4 regulates insulin resistance in trophoblast cells during gestational diabetes mellitus by modulating the miR-6795-5p/PTPN1 axis
Source: J Transl Med. 2022 Apr 21;20:180. doi: 10.1186/s12967-022-03386-8 (PMC9022258; doi:10.1186/s12967-022-03386-8)

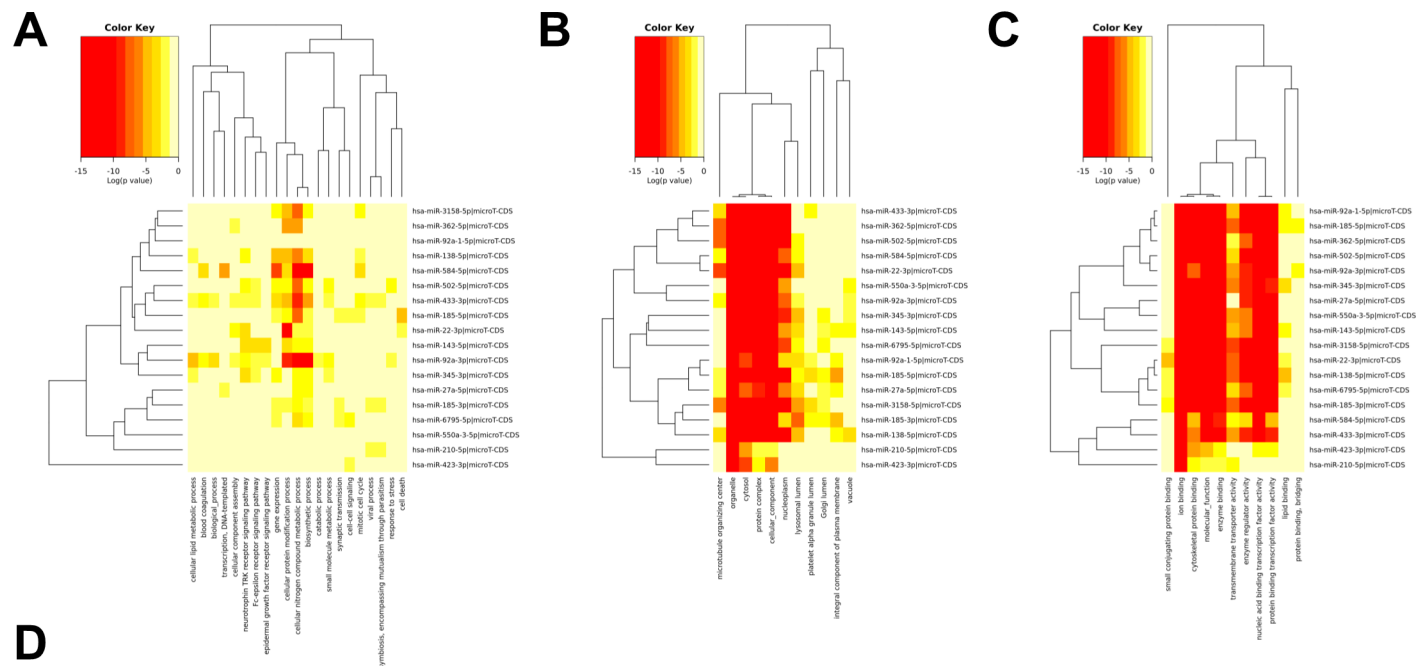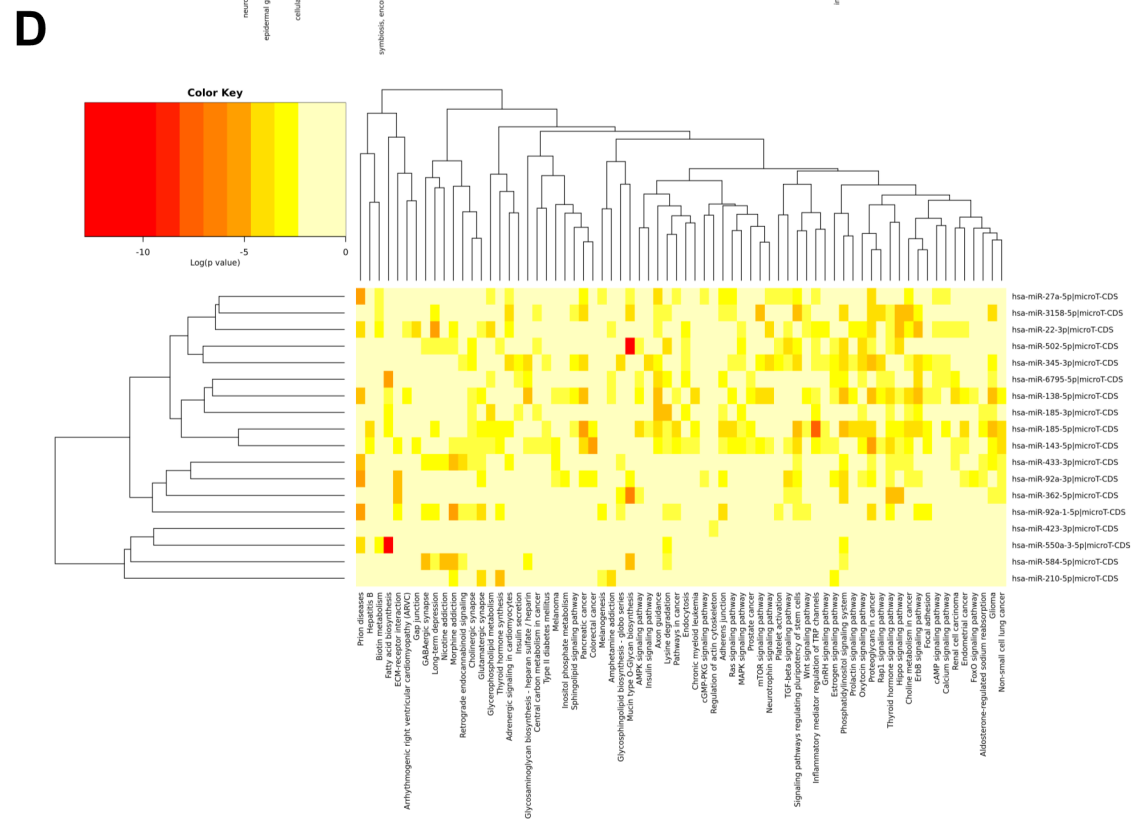

Supplement: Supplementary file 4 — Additional file 4: Figure S1. Enrichment analysis. Cluster diagrams of enriched biological processes (A), cellular components (B), and molecular functions (C) of the GO terms and KEGG pathways (D). The horizontal axis represents the pathway name, while the vertical axis represents the enriched miRNAs. The gradient color represents the log (P-value). [file 12967_2022_3386_MOESM4_ESM.pdf]

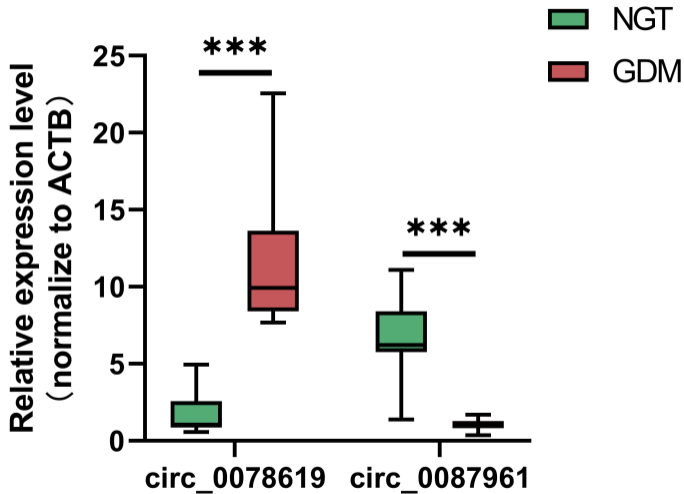

Supplement: Supplementary file 6 — Additional file 6: Figure S3. circ_0078619 and circ_0087961 expression in the gestational diabetes mellitus (GDM, n = 9) and normal glucose tolerance (NGT, n = 9) groups as determined by qPCR. The cell experiment was repeated three times. *** P < 0.001. [file 12967_2022_3386_MOESM6_ESM.pdf]
