# Supplementary material for: circMAP3K4 regulates insulin resistance in trophoblast cells during gestational diabetes mellitus by modulating the miR-6795-5p/PTPN1 axis
Source: J Transl Med. 2022 Apr 21;20:180. doi: 10.1186/s12967-022-03386-8 (PMC9022258; doi:10.1186/s12967-022-03386-8)
Supplement: Supplementary file 5 — Additional file 5: Figure S2. The intersection of of KEGG pathways according to circRNAs function in a miRNA-dependent manner and in a miRNA-independent manner. [file 12967_2022_3386_MOESM5_ESM.pdf]

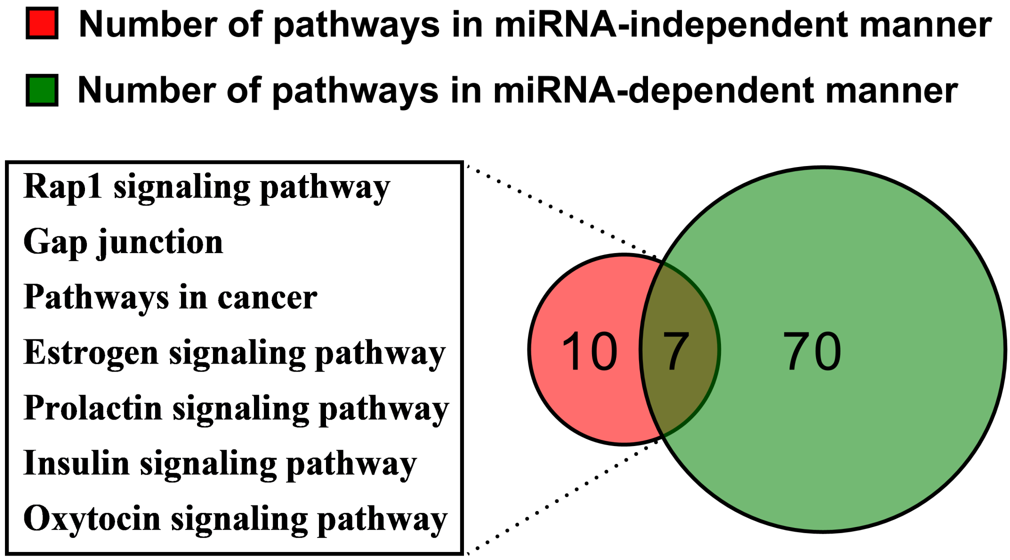
Number of pathways in miRNA-independent manner
Number of pathways in miRNA-dependent manner
Rap1 signaling pathway
Gap junction
Pathways in cancer
Estrogen signaling pathway
Prolactin signaling pathway
Insulin signaling pathway
Oxytocin signaling pathway
10
7
70
